# An Audit of Physical Health Monitoring in the Community Psychiatry Outpatient Setting: Can We Improve?

**DOI:** 10.1192/bjo.2022.392

**Published:** 2022-06-20

**Authors:** Thomas Hall, Gemma Andrews, Darren Carr

**Affiliations:** 1 North Staffordshire Combined Healthcare, Stoke-on-Trent, United Kingdom; 2 Royal Stoke University Hospital, Stoke-on-Trent, United Kingdom

## Abstract

**Aims:**

Care in the community psychiatric setting involves regular monitoring of both mental and physical health. Patients with mental illness worldwide have higher rates of morbidity and earlier mortality, often due to physical disease, most commonly of metabolic or cardiovascular origin. The reasons for these findings are numerous, though a significant contributor is the underperformance of lifestyle screening and subsequent underutilisation of interventions. As standard, it is recommended that practitioners of all grades should, at each appropriate opportunity, assess their patient's current physical status and screen for lifestyle factors that increase risk of morbidity. These include: weekly physical activity, weight/BMI, diet, smoking status and alcohol intake. Our aim was to investigate if our Community Team was meeting both trust-set standards and national standards.

**Methods:**

A list of all outpatient appointments, including all clinic types, and all grades of staff, was generated from 1/11/21 to 19/11/21 giving a total of 48 appointments. A list of questions were then answered using data taken from notes available on an electronic system. This allowed analysis of the frequency of assessment for each lifestyle factor and frequency of offered interventions, where appropriate. Further analysis across all grades of staff, both outpatient appointment clinics and medication monitoring clinics, and across specific mental health disorders was performed.

**Results:**

Each lifestyle factor should have been checked at each appointment and interventions offered where appropriate. In each assessment an intervention could have been offered following identification of a modifiable factor. No factor was assessed at every opportunity. Only 2 interventions (4%) were offered. Targeted Medication Monitoring Clinics (MMC) did not perform better than Outpatient Follow-up Clinics (OPA), OPA offered more interventions. These findings were consistent across all grades of practitioner and diagnoses.

**Conclusion:**

Assessment of modifiable risk factors was not performed at each assessment, and where interventions were appropriate, they were rarely offered. This was a universal issue across the team, and in spite of specialised clinics, or high risk disorders, there was substandard physical health management. Therefore, opportunities to modify risk of physical disease, or improve treatment of the underlying psychiatric disorder are being missed. This is troublesome as community psychiatry often has the space, time, and rapport with patients to explore these issues, furthermore, many psychiatric treatments carry the burden of increased risk of morbidity and mortality. Consequently, the onus should be upon us to manage these risks and improve patient health through simple, short interventions and timely signposting and referrals.

